# *Balaenophilus manatorum* in Debilitated and Bycatch-Derived Loggerhead Sea Turtles *Caretta caretta* from Northwestern Adriatic Sea

**DOI:** 10.3390/vetsci10070427

**Published:** 2023-07-01

**Authors:** Erica Marchiori, Andrea Gustinelli, Viola Vignali, Sara Segati, Simone D’Acunto, Silvia Brandi, José Luìs Crespo-Picazo, Federica Marcer

**Affiliations:** 1 Dipartimento di Medicina Animale, Produzioni e Salute, Università di Padova, viale dell’Università 16, 35020 Legnaro, PD, Italy; federica.marcer@unipd.it; 2 Dipartimento di Scienze Mediche Veterinarie, Università di Bologna, Via Tolara di Sopra 50, 40064 Ozzano dell’Emilia, BO, Italy; andrea.gustinelli2@unibo.it (A.G.); viola.vignali@studio.unibo.it (V.V.); 3 Centro Sperimentale per la Tutela degli Habitat (CESTHA), Via Molo Dalmazia 51, 48122 Marina di Ravenna, RA, Italy; direzione.cestha@gmail.com (S.S.); info.cestha@gmail.com (S.D.); silviabrandi2012@gmail.com (S.B.); 4 Fundación Oceanogràfic de la Comunitat Valenciana, Ciutat de les Arts i les Ciències, C/d’Eduardo Primo Yúfera, 1B, 46013 València, Spain; jlcrespo@oceanografic.org

**Keywords:** ectoparasite, *Caretta caretta*, debilitated turtle syndrome, Adriatic Sea, epibiotic fauna

## Abstract

**Simple Summary:**

Sea turtles, *Caretta caretta*, are hosts for several animal and algal organisms which live on their body surface and develop a symbiotic association with them which can range from mutualistic to parasitic, depending primarily on the species. The copepod *Balaenophilus manatorum* has developed a true parasitic association in that, while exploiting turtle’s skin keratin as a food resource, it may be responsible for cutaneous lesions whenever the equilibrium host-parasite is lost. Studies reporting *B. manatorum* as a component of epibiotic communities of turtles are still sparse in the literature, and little is known about its distribution in the Mediterranean Sea. This study aimed to investigate its presence in *C. caretta* ranging in the Northwestern Adriatic Sea and to investigate the effects of Debilitated Turtle Syndrome (DTS) on the turtle–parasite equilibrium. The results of this study indicate that the copepod is a common epibiont of turtles feeding in this region and that individuals suffering from DTS are more frequently parasitized and have higher copepod burdens. Appropriate attention should be given, in debilitated animals, to proper treatment against external epibionts, taking into account the eventual co-presence of true parasites.

**Abstract:**

*Balenophilus manatorum* (Copepoda: Harpaticoida) is one of the few components of the epibiontic fauna of *Caretta caretta* that show a “true” parasitic association with their host. From rrosive to ulcerative cutaneous lesions may seldom appear as a consequence of the copepod feeding on keratin on turtles’ skin. Debilitating Turtle Syndrome (DTS) is the final outcome of a chronic insufficient assumption of nutrients, generally occurring with the impairment of immune functions and high epibiota burdens. In this survey, the presence of *B. manatorum* in *C. caretta* from the Northwestern Adriatic Sea was investigated and the relation between infection indices and the co-occurrence of DTS was studied. Clinical examination was performed at the time of rescue, including routine hematological assessment; external parasites were isolated mechanically from turtles’ skin and morphologically identified through observation with an optic microscope and SEM. Ten turtles were classified as affected by DTS, all of them being small juveniles with typical clinical and clinicopathological presentation. A higher prevalence, abundance, and density of infection were found in turtles affected by the syndrome. The presence of massive skin coverage by the burrowing barnacle *Pletylepas hexastylos* prevented a proper evaluation of the pathology associated with *B. manatorum* in turtles affected by DTS. In any event, eventual skin damages caused by the parasite may represent a port of entry for secondary infections in such immunocompromised animals. Therefore, infection by *B. manatorum* should not go overlooked in debilitated turtles and should be opportunely treated.

## 1. Introduction

The Northern Adriatic Sea is a well-known neritic feeding ground for loggerhead sea turtles, *Caretta caretta*, in the Mediterranean Sea. Due to the co-existence of a relevant fishing effort in the same area, the region is also known as a hotspot for sea turtle bycatch, especially by trawlers [1]. Although anthropogenic activities represent the greatest threat to the conservation of the sea turtle subpopulation in the region, other health issues, unrelated to human activities, also affect the health status of these animals and are less frequently reported. Debilitated Turtle Syndrome (DTS) is a condition observed in stranded sea turtles all over the world, the most striking aspect of which is the massive epibiont burden which generally covers the skin and carapace of the affected turtles [2]. This clinical presentation is thought to represent the end-stage of starvation, assuming a chronic inadequate intake or absorption of nutrients at the origin [2,3]. In the absence of visible external lesions, the identification of primary causes is somewhat challenging, considering that consequences of the debilitation also occur simultaneously, overlapping and worsening the precedent condition. Insufficient food intake may be the consequence of simple food unavailability, or the impossibility to ingest food (e.g., obstruction of the digestive system, such as ingestion of foreign bodies, including hooks and lines). Any condition preventing normal swimming and predatory behaviour may virtually result in improper feeding, including traumatic lesions, entanglement, and cold stunning. Intestinal and metabolic diseases have also been reported as causes of the insufficient absorption of nutrients [3]. Though causes may widely differ, the consequences of debilitation are almost always consistent, resulting in common clinical signs and symptoms. High burdens of epibiota are an almost constant finding in debilitated sea turtles [2,4,5,6]. Excessive barnacle loads have been attributed to the inability of turtles to perform normal “self-grooming” behaviour, such as rubbing against objects, or accessing “cleaning stations,” in which specific omnivore fish species offer the mutual advantage of feeding on turtles’ epibionts while helping in the control of their burden [7,8]. Surface drag caused by the overgrowth of barnacles further decreases the ability of affected turtles to swim properly and feed, especially when their eyes and mouth are covered by sessile epibionts, and increases energy expenditure for performing normal movements [9]. Therapy generally focuses on rehydration, nutritional support, and the elimination of infectious diseases, along with the underlying causes, when identifiable.

Harpacticoid copepods are an important component of the meiofauna, including about 3000 known species that widespread in marine and freshwater ecosystems [10], and it is estimated that a great part of the diversity within this family is still overlooked [11]. Only five harpacticoid species live in association with vertebrate species, with the genus *Balaenophilus*, the most widespread, exploiting marine mammals and sea turtles as living substrates. The type of association developed with the vertebrate host has been a controversial topic for a long time. Evidence of a “true” parasitic association of *Balaenophilus manatorum* (previously *Balaenophilus umigamecolus*) with the sea turtle host has been provided by Badillo et al. (2007) [12] and Crespo-Picazo et al. (2017) [13], who demonstrated the ability of the species to feed on the keratin of the host skin, seldomly causing erosive to ulcerative lesions. Most severe disease has been described in captive hatchlings, with the underdeveloped immune system being cited as one of the predisposing factors for high parasitic loads [13].

In the present paper, we aimed to (i) report the presence of *B. manatorum* in loggerhead sea turtles from the Northern Adriatic Sea and (ii) study the relationship between infection by *B. manatorum* and the co-presence of DTS, which is diagnosed in turtles by combining clinical and clinicopathological findings.

## 2. Materials and Methods

### 2.1. Clinical Data Collection

**Figure 1:**
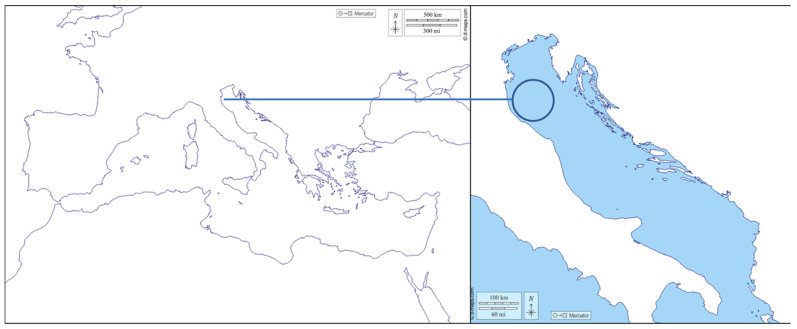
Sampling site in the Northern Adriatic Sea, embracing Ferrara and Ravenna provinces.

Twenty-four loggerhead turtles admitted to the rescue facility run by Centro Sperimentale per la Tutela degli Habitat (CESTHA) in Marina di Ravenna, between June and October 2021, were included in this study. Of these, 10 animals were found stranded along the shoreline or floating in the waters off the provinces of Ferrara and Ravenna (Figure 1). The remaining 14 turtles were rescued after being bycaught by trawlers in the corresponding offshore marine area. Water temperatures registered in the whole sampling period ranged from 20 °C to 27 °C.

At admission, all turtles were submitted to clinical assessment, including physical examination and routine blood work (i.e., haematocrit and total protein estimate, and qualitative and quantitative white blood cell evaluation). Biometric data, including curved carapace length (CCL), notch-to-tip, and body weight were collected at the time of entry. Neurological examination was performed with the animals out of water and their ability to dive and surface was evaluated in water tanks after stabilization by a technician or a veterinarian. Nutritional condition was assessed always by the same veterinarian observing the axillary, inguinal, and neck regions, plastron concavity, and orbits, and assigning a Body Condition Score (BCS) from 1 to 5, ranging from cachectic to optimal condition [14]. External examination was conducted to exclude the presence of traumatic injuries and to evaluate the percentage of body surface covered by sessile epibiota. This was conducted by visual examination both immediately at the time of arrival by technicians on site and by taking pictures of the animals on dorsal and ventral recumbency which were later re-examined. A blood sample (at least 1 mL) was withdrawn from the cervical sinus of the jugular vein into lithium-heparin tubes after surgical preparation of the site. Hematocrit (HCT) and total protein (TP) concentration were obtained with standard centrifugation in microhematocrit capillary tubes; plasma was evaluated using a refractometer. In order to evaluate the immune status of the turtles, fresh blood smears were prepared, air-dried, and stained with Diff Quick for differential white blood cell count and observation of blood cell morphology [15,16]. Total white blood cell count was estimated with a Neubauer haemocytometer after dilution to 1:100 with Natt and Herrick’s staining solution (Bioanalytic GmbH, Umkirch, Germany). Ranges for the evaluation of HCT and TP, and for total WBC count, were compared with Casal and Orós (2009) [17]. All clinicopathological analyses were conducted by a trained veterinarian.

### 2.2. Parasitological Analyses

In order to detect *B. manatorum*, turtles were gently scrubbed with a nylon soft brush and washed with tap water soon after stabilization (i.e., within some hours from recovering normal surfacing behaviour), before entering rehabilitation tanks. Soft skin was also brushed to remove non-sessile external parasites. Washes were collected and filtered with a 0.5 mm sieve. All material kept on the filter was preserved in 70% ethanol flasks until examination at the Fish Pathology Unit of the Department of Veterinary Medical Sciences of Bologna University. For each sample, copepods were collected from washes at the stereomicroscope and counted. After isolation, morphometric characteristics of randomly selected specimens from each sample were studied under an optic microscope by NIS Elements D software (Nikon) (*n* = 21) and Scanning Electron Microscopy (SEM) (*n* = 5) to confirm species identification. Samples were examined by SEM in order to describe some morphological features of larval and adult stages. The specimens were postfixed in 1% osmium tetroxide in cacodylate buffer, dehydrated through a graded ethanol series, critical point dried, sputter-coated with platinum, and observed using a JEOL JSM 6700F SEM (Basiglio [MI], Italy) operating at 5.0 kV according to the process described in Tedesco et al. (2018) [18].

Morphometric characteristics of copepods were compared with the descriptions in the literature for species identification [19,20]. Similarly, specific key features were used for the identification of sessile epibionts [21].

### 2.3. Data Analysis

Prevalence (percentage of positive turtles), abundance (number of parasite individuals per turtle), and intensity values (number of parasite individuals per infected turtle) for *B. manatorum* were displayed using simple descriptive statistics. Since formulae for the calculation of body surface area in chelonians are lacking in the literature, weight was taken as a proxy of body surface area to calculate parasite density [22,23], assuming these two parameters to be positively correlated.

In order to explore any influence of debilitated turtle syndrome on the aforementioned epidemiological indices, turtles were classified as affected (DTSt) when presenting with the following findings: depressed sensorium associated with anamnesis of stranding or floating offshore, BCS < 3, extensive epibiota coverage (>50% percentage of body surface) or barnacles covering eyes or rhamphotheca, no evidence of traumatic injuries, anemia, and hypoproteinemia [2]. Animals deviating from this presentation, lacking two or more of the above-mentioned findings, were classified as non-affected (non-DTSt). Fisher’s exact test and Mann–Whitney test were used for the comparison of prevalence and abundance, intensity, and density between the two groups. Clopper–Pearson (exact) method was used for 95% CI definition. Student’s T test was used to compare HCT, TP, and total WBC values in DTSt and non--DTSt to assess the reliability of these values as markers for DTS in our sample.

Statistical tests were conducted on the online software Quantitative Parasitology QPweb [24]. Level of significance for all tests was set at *p* < 0.05.

## 3. Results

### 3.1. Clinical Data

**Figure 2:**
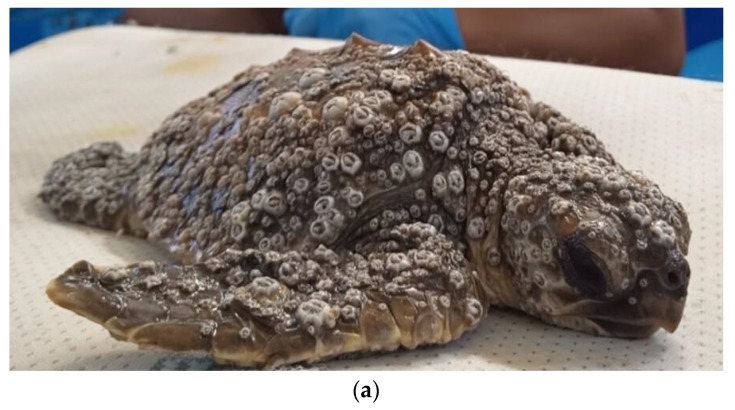

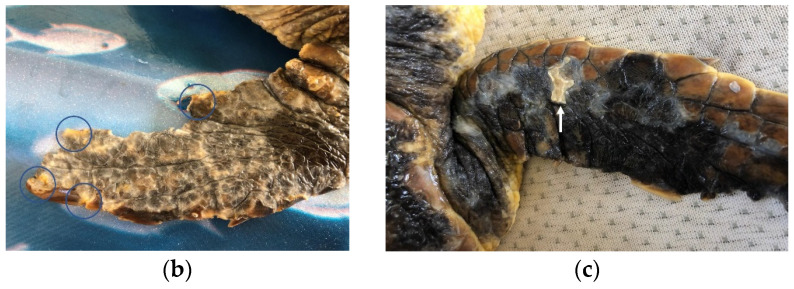
Clinical presentation at arrival and cutaneous lesions on flippers of loggerhead sea turtles affected by DTS after barnacle removal. (**a**) Barnacle *P. hexastylos* covering most parts of body surface appearing as the almost exclusive barnacle species. (**b**) Diffuse erosive and multifocal ulcerative lesions (circles) on the fore-flipper surface; (**c**) deep ulcerative lesions (arrow) on the dorsal aspect of a fore-flipper.

Overall, 10 turtles out of the 24 were classified as affected by DTS, with all being small juveniles (mean CCL 18.6 cm, range 14.5–22.5 cm) and all but one being found stranded along the shore. One turtle was recovered while floating offshore, showing no voluntary attempts to escape at catching. At neurological examination, four turtles were severely depressed, with little or no response to lifting, five animals were classified as depressed, and one turtle as slightly depressed, showing a reduced but timely response to all stimuli. A BCS of 2/5 was attributed to all turtles of this group, which showed muscular atrophy, sunken eyes, and inguinal and axillary fossae from normal to slightly depressed. Barnacles of the species *Platylepas hexastylos* were found covering ≥50% of turtles’ skin, preferentially distributed on soft skin areas (axillary and inguinal regions and flippers), but were also abundant on the carapace, plastron, and head (Figure 2a). Occasionally, the eyelids were also affected. No other barnacle species were observed in this group. The detachment of *P. hexastylos* after freshwater baths revealed erosive to necrotic cutaneous lesions, and tissue loss along the margins of the extremities (Figure 2b,c). None of the turtles showed other external injuries (e.g., traumatic). Anaemia was classified as severe in eight animals (HCT range 8–12%) and moderate in two (14–16%). Hypoproteinaemia was registered in 5/10 animals (TP < 2.0 g/100 mL), leucocytosis in 1 individual (tot WBC > 19 × 10^3^/µL) and heterophil toxicity of different degrees was observed in 3 individuals. The lymphocyte relative count ranged from 1% to 17% (Table 1 and Table S1).

NonDTS turtles (*n* = 14) were all rescued from bycatch events in trawlers. The group included juvenile to adult animals, with CCLs ranging from 21 to 91 cm (mean 53.1 cm). Depressed mentation was observed in 6 out of 14 animals at admission, with the remnant being normally reactive to stimuli. The body condition scores ranged from two to four. The percentage of body surface covered by barnacles ranged from 0% to 30%, with *Chelonibia* sp. being the most frequently encountered barnacle species, preferentially fixed on the carapace. The HCT ranged within normality (26–46%), as did total protein (>2.2 g/100 mL). No heterophil toxicity was observed in the blood films. The total WBC count ranged within normality, except for one case of leucocytosis that probably attributable to stress leukogram and was not associated with clinical disease.

### 3.2. Parasitological Examination

Adult copepods were detected in 17/24 of the turtles. All of the specimens (*n* = 1803) were identified as *Balaenophilus manatorum* from morphology. The overall mean intensity of infection was 106.5, with a range from 3 to 377 individuals per turtle. The descriptive indices of infection in the overall sample and in DTSt and non-DTSt are reported in Table 2.

**Figure 3:**
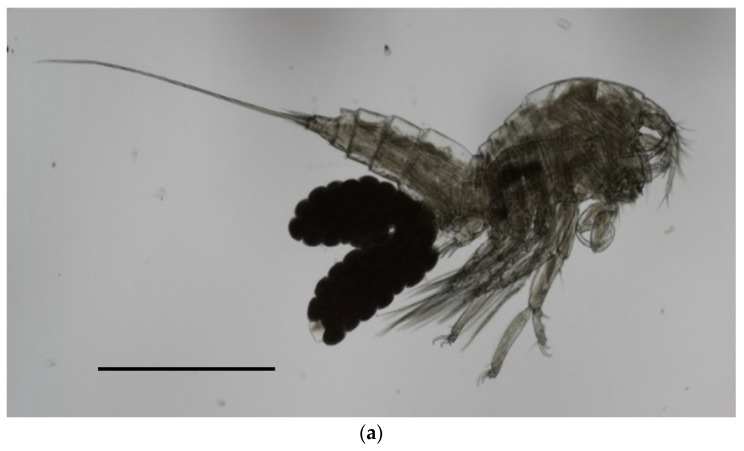

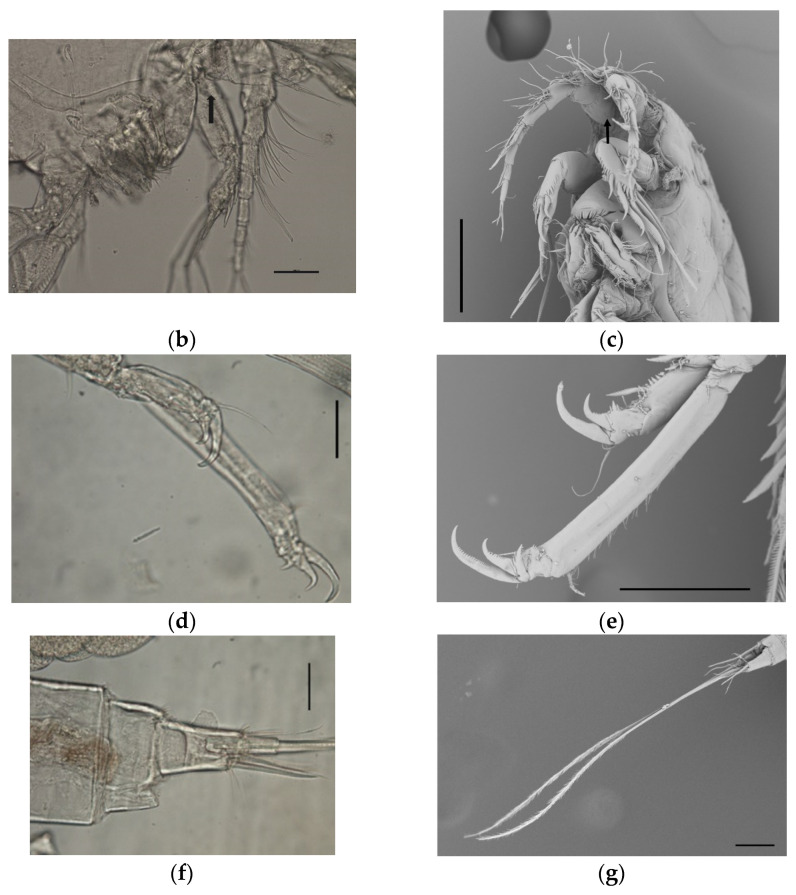
Microphotographs of individuals of *B. manatorum* collected from *Caretta caretta* from the Northern Adriatic Sea using optic microscope (left side) and SEM (right side). (**a**) Full-body image of an adult female. (**b**,**c**) A coronula of spines is visible on the inner face of the antennula allobase (black arrow); (**d**,**e**) Leg 1 showing three hooks on the endopode and exopode; (**f**) caudal rami short; (**g**) terminal setae. Scale bars: (**a**)—500 µm; (**b**,**d**,**f**)—50 µm; (**c**,**e**,**g**)—100 µm.

In detail, the morphological key features observed in our sample were the following: average body lenght ± sd (measured in µm) in females 1111.5 ± 61.6 (range 999.5–1189.46) (Figure 3a) and 1017 ± 79.8 (range 863.6–1133.5) in males; antennula made up of nine segments, with setae of variable numbers on segments 2–9; antenna with a short coxa, allobase carrying a coronula of spines on the inner side (Figure 3b,c), and a small exopod made up of two segments, having one and two setae, respectively; endopod of antenna carrying, overall, seven claws and three geniculate; maxilliped made up of a short syncoxa, having two setae at the distal end, a longer basis with two short setae at midlength, and a distal, hook-shaped endopod carrying two, very fine setae on the inner side; leg 1 having three small claws on both the endopod and exopod (Figure 3d,e); caudal rami being short (Figure 3f), with two elongated setae (average length 811.3 ± 56.7 in females, 831.1 ± 69.7 in males) (Figure 3g). No setae were observed on segment 2 of the exopode of leg 4.

A higher prevalence, abundance, and density of infection were found in the DTSt group compared to the non-DTSt group. The differences in intensity approximated significative values (Table 2). Clinical and parasitological data of the single individuals are reported in Table S1.

## 4. Discussion

This study aimed to detect the copepod *B. manatorum* in sea turtles from the Adriatic Sea and relate infection indices with clinical and clinicopathological findings. Turtles affected by DTS that were included in the study were all juvenile specimens stranded along the north-western coast of the Adriatic Sea, which appeared to have a higher risk for hosting the copepod and have a higher burden with respect to turtles coming from bycatch. *B. manatorum* apparently has a worldwide distribution, as this species has been reported on the Eastern and Western Pacific coasts [20,25] as well in the Western Mediterranean Sea [12,13,26]. Previous surveys that were conducted on the epibiota of *C. caretta* in the Adriatic Sea [27,28], as well as in the Central [29,30] and Eastern Mediterranean [31], did not adopt specific methods for the detection of *B. manatorum*, and, consequently, no earlier reports of the species are present from these regions. As Domènech et al. (2014) [32] pointed out, the detection of this tiny ectoparasite is subordinated to the application of proper research methods, i.e., the use of a preliminary wash and filtering through a small mesh sieve, which was not included in the methodology followed by the aforementioned studies. Recently, Karaa et al. (2019) [33] reported the presence of one specimen of Harpacticoida on the carapace of one loggerhead turtle from the Tunisian coast, suggesting its identification as *Balaenophilus* sp., but the methods for epibiont collection were not disclosed in that study. This finding further suggests a pan-Mediterranean distribution of *B. manatorum* in loggerhead turtles. Data on the prevalence of infection in free-ranging loggerhead sea turtles have only been provided for the area of the Western Mediterranean (82.7%) [12]. Our data suggest a potentially comparable prevalence in loggerhead sea turtles from the Adriatic Sea (70.8%), but given the limited sample herein considered, caution is warranted when considering this value. The existence of different sibling species of *B. manatorum* in the Mediterranean should not be ruled out, considering separate spatial distribution [19], at least until a complete molecular study is conducted on the isolates.

A higher prevalence and intensity of infection by macro and microparasites are generally expected in animals suffering from depression of their immune functions, following alteration of the equilibrium between the host and its parasites [34]. Defects in the activity of lymphocytes have been reported in debilitated turtles, which finally results in the suppression of the humoral immunity in these individuals [2]. Suppression of the adapted immune response is a general consequence of extreme energy restrictions during starvation in mammals [35,36], and similar effects are expected in reptiles. Innate immunity apparently activates as compensation for this deficit in sea turtles to provide immediate defence against bacterial and parasitic infections [2]. Although no specific evaluation of immune functions was conducted in this study, and the lymphocyte relative counts were in some cases overlapped between individuals of the two groups, there was a significative trend in DTSt of have lower lymphocyte counts, which is correlated with lower immune defence, and the presence of toxic heterophils in this group supports the hypothesis of underlying inflammation and the compensatory activation of innate immunity [2,15]. Underdevelopment of the immune system was indeed suspected to be one of the co-factors inducing severe lesions by *B. manatorum* in hatchling loggerheads by Crespo-Picazo et al. (2017) [13], along with stress from captivity and high water temperatures. Similarly, high temperatures may also have increased the recruitment rate of *B. manatorum* in our study, as all animals were sampled in the warmer period of the year. Future studies should assess the impact of seasonal temperature variation and other abiotic factors on infection prevalence and intensity. The softer skin of hatchlings and small juveniles may also have facilitated the infection by *B. manatorum* in the study by Crespo-Picazo et al. (2017) [13] and in our study as well. Definite conclusions are not possible, given the overlap of young age and immune deficiency in both studies. Appropriate prophylactic and therapeutic measures, especially, should be adopted when dealing with small juveniles in captivity.

The facilitation of *B. manatorum* colonization in DTSt group may have also been offered by the previous overgrowth of *P. hexastylos* on the turtles’ body surface. Although the temporal succession of epibiont communities on sea turtles hosts is still poorly understood, it is thought that hard, sessile species, such as barnacles, are the first to colonize turtle carapaces; these “pioneer species” [37] generate new niches for colonization by motile epibionts, facilitating their settlement to turtle carapace [38,39,40]. Lazo-Wasem et al. (1997) [25] also found the co-occurrence of *B. manatorum* with the sessile barnacle *Stomatolepas praegustator* to not be coincidental and hypothesized that *B. manatorum* may feed on irritated skin close to the embedded barnacles. Specialized hooks and claws likely allow adult and immature *B. manatorum* to grasp tightly to turtles’ skin, independently from the presence of other epibiotic species, as the motile amphipod *Caprella* spp. does; both species, indeed, can be found as an exclusive component of the epibiotic community of turtles [13,38]. High burdens of *B. manatorum* were isolated from one turtle of the non-DTSt group in which the overgrowth of algae was also observed, covering the caudal midpart of the carapace. Further studies should test whether colonization by animals and/or algal epibionts facilitates the recruitment of *B. manatorum.*

The higher prevalence of *P. hexastylos* in DTSt vs. *C. testudinaria* is difficult to interpret. Both species are described in *C. caretta* in the Adriatic Sea [28]. *P. hexastylos* was thought to be a useful indicator of habitat use, being more typical of turtles floating in pelagic areas [30]. Ten et al. (2019) [41] recently reported this species to have a limited value as a habitat indicator, as it has been detected in both oceanic and neritic turtles with similar abundance, but has been negatively correlated with the abundance of *C. testudinaria*. The soft carapace skin of juveniles may have facilitated the recruitment all over the body surface of the burrowing barnacle *P. hexastylos*, whose presence is generally limited to the skin of throat, axillary areas, and flanks, finally competing with *C. testudinaria* for its niche. A comparison with subadult, DTS-affected loggerhead sea turtles should be conducted to further explore this issue. Meanwhile, conclusions regarding this aspect sill remain speculative.

The high loads of the barnacle *P. hexastylos* in the DTSt group prevented a proper evaluation of the pathological effect of the copepods on turtles’ skin. Erosions and ulcers were evident on all body surfaces of the turtles at the time of barnacle removal, with most severe lesions being represented by areas of necrotizing ulcerative dermatitis, mostly on soft skin areas (neck and flippers). Barnacles of the species *P. hexastylos* are listed among the “burrowing epibionts,” here indicating a varied group of organisms whose bodies can become embedded within both the hard and soft tissues of host turtles; the opposite, non-burrowing barnacles (e.g., *Chelonibia* spp.) simply fix their bodies on the epidermis surface with no tissue penetration [38]. Although *P. hexastylos* is found technically external to the skin, as a thin epidermal layer remains intact under their bodies, at the time of their detachment, erosions and ulcerative lesions can become evident, as well as the loss of the external beta-keratin layer [42,43]. Similar erosive lesions were extensively present in the DTSt group, but their appearance strikingly overlaps the lesions caused by *B. manatorum*, as reported in the literature [12,13,20]. As an exception among marine symbionts, indeed, *B. manatorum* has been demonstrated to be able to feed on keratin, exploiting the skin of manatees and sea turtles as optimal substrates [12]. Discoloured or yellowish areas are described on the skin of turtles as the result of keratin consumption by the copepods, mostly on soft skin areas, and occasionally on the carapace and plastron [12,20]. At histology, ulcerative necrotizing dermatitis involving the superficial dermal layer is reported in severe cases, as well as dyskeratosis and inflammation of varying degrees [12,13]. In this study, no biopsies were conducted to detect the presence of copepods within the lesions. Therefore, it still remains to be ascertained to what extent the copepods may have contributed to altering the skin barrier in the DTS-affected turtles, potentially favouring secondary bacterial infections in immunodeficient animals. The mean intensity of infection in turtles with lesions is variable in the literature but higher than ours in the study by Crespo-Picazo et al. (2017) [13] (337.4 copepods per turtle). Badillo et al. 2007 [12] reported neither gross nor microscopic tissue alterations in turtles infected with 113 copepods, and only inflammatory reactions were evident at histopathology in turtles with from 585 to 1193 specimens. Nevertheless, the size of the turtles was not known in this latter study, making it difficult to compare with our study.

A mass stranding event of juvenile loggerhead turtles was reported along the same North-Western Adriatic coast in 2009 [44]; comparably to the present case, the stranding of such juvenile turtles happened in the same season—from late July to September—and involved only small individuals, with CCL ranging from 17 to 25 cm. Massive infestation by *P. hexastylos* was reported in those specimens as well, covering the skin and mucosae; similar clinicopathological findings were reported [45], but sampling for *B. manatorum* was not performed. The reasons for the unusual stranding of small juvenile *C. caretta* in the area remain to be ascertained; specimens of this size represent an unusual finding in the region, which is mainly populated by large juveniles, and subadult and adult *C. caretta* at their neritic phase. The shallow waters of the Northern Adriatic Sea probably represent an unsuitable feeding ground for juveniles in their pelagic life-stage, and may have led to the development of DTS, which would also be confirmed by the almost exclusive presence of algae in their feces for days after rescue (Segati, pers. Comm.), as also reported ain 2009 [45], which are an uncommon food item for loggerheads at this ontogenic stage. Prompt supporting therapy is mandatory for the recovery of debilitated turtles; repeated freshwater baths are administered for progressive barnacle removal and may be resolutive for infection by *B. manatorum* as well [13].

## 5. Conclusions

*B. manatorum* is a component of the epibiotic communities of loggerhead sea turtles in the Northern Adriatic Sea. Studies on the real prevalence in free ranging turtles are encouraged in the area in order to increase our knowledge on epibiotic communities’ composition in *C. caretta*, and especially for that part of the epibiota which is considered, per definition, parasitic. Turtles suffering from DTS and, more generally, those carrying high burdens of burrowing barnacles, should be carefully evaluated for the presence of *B. manatorum* and opportunely treated.

## Figures and Tables

**Table 1:** Main clinicopathological values in loggerhead turtles in this study. Statistical comparison between DTSt and non-DTSt values is shown. Student’s T test results are given for the comparison between the two groups. HCT, hematocrit; TP, total protein; WBC, total white blood cells; lymph%, percentage of lymphocyte in white blood cell formula; het tox, toxic heterophils as evaluated from blood smear. * Statistically significant. All values are presented as mean ± standard deviation.

	HCT (%)	TP (g/dL)	WBC	Lymph %	Het Tox
DTSt (*n* = 10)	11.2 ± 2.57	1.73 ± 0.59	10975.19 ± 4017.99	11.55 ± 7.58	0–3+
non-DTSt (*n* = 14)	34.53 ± 8.06	3.87 ± 1.04	8540.2 ± 4688.8	19.14 ± 7.54	0
*p*	<0.01 *	<0.01 *	>0.05	0.028 *	

**Table 2:** Epidemiological indices in the overall sample considered and in turtles affected and non-affected by DTS. *p*-values refer to the comparison between the latter two groups, obtained with Fisher’s exact test for prevalence values and Mann–Whitney test for intensity, abundance, and density. P%, prevalence (95% confidence interval calculated with Clopper Pearson exact method); medI, median intensity; medA, median abundance; medD, median density; IQR, interquartile range. * Statistically significant.

	Overall	DTSt	non-DTSt	*p*
P (%) (95% CI)	70.8 (48.9–87.4)	100 (69.1–100)	50. 0 (23.0–76.9)	0.010 *
medI (IQR)	47 (13.0–170.7)	140.5 (54.5–174.2)	13 (8.5–22.7)	0.051
medA (IQR)	19.5 (0.0–91.7)	140.5 (54.5–174.2)	14.5 (0.0–12.0)	0.001 *
medD (IQR)	0.37 (0.0–6.3)	8.62 (4.1–11.4)	0.02 (0.0–0.3)	0.001 *

## Data Availability

Dataset providing information supporting results of this study is reported in Supplementary materials.

## Supplementary Materials

The following supporting information can be downloaded at: https://www.mdpi.com/article/10.3390/vetsci10070427/s1, Table S1: Individual clinical and parasitological data of loggerhead sea turtles included in the study.
